# A High-Quality Reference Genome for the Invasive Mosquitofish *Gambusia affinis* Using a Chicago Library

**DOI:** 10.1534/g3.118.200101

**Published:** 2018-04-27

**Authors:** Sandra L. Hoffberg, Nicholas J. Troendle, Travis C. Glenn, Ousman Mahmud, Swarnali Louha, Domitille Chalopin, Jeffrey L. Bennetzen, Rodney Mauricio

**Affiliations:** * Department of Genetics; † Department of Environmental Health Science; ‡ Institute of Bioinformatics, University of Georgia, Athens, GA 30602; § Department of Computational Biology; ** Department of Oncology, St. Jude Children’s Research Hospital, Memphis, TN, 38105

**Keywords:** *Gambusia affinis*, Dovetail Genomics, Poecilid, whole genome sequencing, *de novo* assembly

## Abstract

The western mosquitofish, *Gambusia affinis*, is a freshwater poecilid fish native to the southeastern United States but with a global distribution due to widespread human introduction. *Gambusia affinis* has been used as a model species for a broad range of evolutionary and ecological studies. We sequenced the genome of a male *G. affinis* to facilitate genetic studies in diverse fields including invasion biology and comparative genetics. We generated Illumina short read data from paired-end libraries and *in vitro* proximity-ligation libraries. We obtained 54.9× coverage, N50 contig length of 17.6 kb, and N50 scaffold length of 6.65 Mb. Compared to two other species in the Poeciliidae family, *G. affinis* has slightly fewer genes that have shorter total, exon, and intron length on average. Using a set of universal single-copy orthologs in fish genomes, we found 95.5% of these genes were complete in the *G. affinis* assembly. The number of transposable elements in the *G. affinis* assembly is similar to those of closely related species. The high-quality genome sequence and annotations we report will be valuable resources for scientists to map the genetic architecture of traits of interest in this species.

Mosquitofish (*Gambusia affinis*) are small freshwater fish in the Poeciliidae family that are native to the southeastern United Stated and northern Mexico and live on the banks of streams or lakes. Many species in the Poeciliidae have been used as models for a broad range of evolutionary and ecological studies. For example, since the poeciliids are viviparous, they have been studied for the evolution of the placenta and viviparity (Pollux *et al.* 2009). Related fish in the Poeciliidae family have been used to study life history evolution (Reznick and Endler 1982), phenotypic plasticity (Trexler and Travis 1990), adaptations to extreme environments (Tobler *et al.* 2006; Tobler *et al.* 2007), sex-chromosome evolution (Lamatsch *et al.* 2000), sex determination systems (Volff and Schartl 2001), sexual selection (Basolo 1990), genital evolution (Langerhans *et al.* 2005), biological invasions (Sakai *et al.* 2001) and social behavior (Constanz 1975; Farr 1980).

Since the early 1900s, the mosquitofish has rapidly expanded from its native range in North America and is now considered one of the most widespread freshwater fish in the world (Pyke 2008). It was purposely introduced into every continent besides Antarctica (Krumholz 1948; Lever 1996) to control mosquito populations during malaria and yellow fever outbreaks (Howard 1920; Howard 1910) (although it is not especially effective for this purpose; Lloyd *et al.* 1986), and has led to declines in native insect, fish, and amphibian populations in its introduced range (Pyke 2008).

The creation of a high-quality genome assembly for *G. affinis* will provide an important resource to study the genetic basis of traits commonly studied in Poeciliidae (described above). Because *G. affinis* is highly invasive, a high-quality genome will also allow us to understand the genetic basis of traits that significantly differ between invasive and non-invasive species and can help us to understand how invasiveness evolves. Currently, few members of the Poeciliidae family have sequenced genomes, including platyfish and swordtails in the genus *Xiphophorus* (Schartl *et al.* 2013; Shen *et al.* 2016), as well as the guppy, *Poecilia reticulata* (Fraser *et al.* 2011). A sequenced genome in the *Gambusia* genus will provide a sequenced genome in the third major clade in this family and facilitate genomic and phylogenetic comparisons between *Xiphophorus*, *P. reticulata*, and *G. affinis*, and could be used as a reference genome for comparative genomic studies in diverse fields.

We created a high-quality genome assembly of *G. affinis* using Illumina sequencing of both traditional paired-end libraries and an *in vitro* proximity-ligation Chicago library (Putnam *et al.* 2016). The Chicago library method in conjunction with the HiRise software pipeline is designed to bridge gaps in alignment due to repetitive sequences (Putnam *et al.* 2016) and thereby increase assembly contiguity by up to twenty-fold, despite only using short read data from the Illumina platform. After the creation of a high-quality genome, we annotated this genome using genic sequences from *X. maculatus* and *P. reticulata*, and compared the gene and repetitive element content and quality of the *G. affinis* genome and assembly to related fish species.

## Methods

### Library preparation and de novo shotgun assembly

We constructed our *de novo* genome assembly for *G. affinis* using a short insert library of DNA (Fig S1). We collected a single male fish from the Zuibaiji River in Japan (located at the GPS coordinates: 33.59111, 130.25444) in 2010 and stored this fish in 70% ethanol until we extracted DNA from the muscle tissue using a phenol-chloroform extraction. DNA was stored in TE until library preparation. A male fish was used because males possess homomorphic sex chromosomes (Volff and Schartl 2001).

We sheared the genomic DNA using the Covaris S2 (Covaris, Woburn, MA, USA) targeting a 600bp average fragment size. The sheared DNA was end-repaired, adenylated, and ligated to TruSeq LT adapters using a TruSeq DNA PCR-Free Library Preparation Kit (Illumina, San Diego, CA, USA). We purified the ligation reaction using a Qiaquick Gel Extraction Kit (Qiagen, Venlo, The Netherlands) from a 2% agarose gel. We sequenced the library on an Illumina HiSeq 2500 at the University of California at Los Angeles Genotyping and Sequencing Core to obtain paired-end (PE) ∼100 bp reads.

Reads were sent to Dovetail Genomics (Santa Cruz, CA, USA) for construction of a draft genome assembly using Meraculous v. 2.0 (Chapman *et al.* 2011). We used default parameters when available with a kmer size of 31bp for *de novo* assembly, and ignored kmers with a depth less than 3. We compared the kmer depth on trimmed reads using Meraculous’ mercounting and untrimmed reads using Dovetail’s mercounting software, which fully tallies very high copy counts. The bubble_depth was set to 20 because that was near a local minimum average depth of kmer coverage (21; Fig S2) on bubbletigs (unique-extension contigs extended by popping “bubbles” caused by SNPs). Reads were trimmed for quality and sequencing adapters using Trimmomatic (Bolger *et al.* 2014). This produced a 594.6 Mbp assembly, with scaffold N50 of 31 kbp and contig N50 of 13.9 kbp.

### Chicago library prep and scaffolding the draft genome

To improve the *de novo* assembly, we created a Chicago library (Putnam *et al.* 2016) at Dovetail Genomics. This required extracting DNA from another sample to obtain higher molecular weight DNA. Briefly, ≥ 0.5 μg of high molecular weight genomic DNA was extracted from muscle of a male *G. affinis* from the Platt River in Nebraska (located at the GPS coordinates: 40.8379, -96.7072) as in Allen *et al.* (2006) with modifications made to the extraction buffer, incubation time, and temperature by Dovetail so that the mean DNA fragment size was ∼50 kbp. The DNA was further purified using a Qiagen Genomic column. Chromatin was reconstituted *in vitro* onto naked DNA, and fixed with formaldehyde. Fixed chromatin was digested with *Dpn*II, resulting 5′ overhangs were filled in with biotinylated nucleotides, and free blunt ends were ligated together. After ligation, crosslinks were reversed and DNA was purified from protein. Biotin that was not internal to ligated fragments was removed from DNA, DNA was sheared to a mean fragment size of ∼350 bp, and sequencing libraries were generated using NEBNext Ultra enzymes (New England Biolabs, Ipswich, MA, USA) and Illumina-compatible adapters. Biotin-containing fragments were isolated using streptavidin beads before PCR enrichment of the library. The Chicago library was sequenced on an Illumina HiSeq 2500 at Dovetail Genomics to obtain PE∼100 bp reads.

The *G. affinis* draft genome in FASTA format, shotgun sequences, and Chicago library sequence (57M read pairs; PE∼125 bp) in FASTQ format were used as input data for HiRise (Putnam *et al.* 2016). HiRise is a software pipeline designed specifically for using Chicago library sequence data to assemble genomes. We aligned the shotgun data and Chicago library sequences to the draft input assembly using a modified SNAP read mapper (http://snap.cs.berkeley.edu). Shotgun data were used to detect regions of the assembly with abnormally high coverage, which were omitted when scoring joins and breaks. We analyzed the separations of Chicago read pairs mapped within draft scaffolds by HiRise to produce a likelihood model, and used the resulting likelihood model to identify putative misjoins and score prospective joins. Then we used scaffolding and shotgun sequences to close gaps between contigs that were not in the same scaffold in the draft input assembly in Meraculous’s gap-closing “marauder” component. In this way, HiRise uses the Meraculous feature to close the gaps it creates when making joins.

### Gene prediction and annotation

We used the MAKER genome annotation pipeline (Campbell *et al.* 2014) to identify the locations of *G. affinis* genes. MAKER combines several classes of data, including RNAseq data or proteins from closely related species, to generate *ab initio* gene predictions. The MAKER pipeline consisted of the following steps: 1) RNAseq and protein sequences from *X. maculatus* (Schartl *et al.* 2013) and *P. reticulata* (Fraser *et al.* 2011) were used for the initial annotations; 2) the initial annotations were used to train SNAP gene prediction tool (Korf 2004) multiple times; and 3) the final set of gene annotations were generated from the trained *ab initio* SNAP predictions (Files S1-S5).

To assess the quality of our *G. affinis* gene annotations, we used BLAST to compare the number of gene annotations in *G. affinis* to those in *X. maculatus*, *P. reticulata*, and *Oryzias latipes* (medaka; Kasahara *et al.* 2007) by setting an e-value cutoff of 10^−10^ (File S6). These species were chosen because *X. maculatus* and *P. reticulata* are Poeciliidae with fully sequenced genomes and *O. latipes* has a high-quality genome sequence that is often used for comparisons in fish species. To functionally annotate *G. affinis* genes, we identified the best homologs from the UniProt/Swiss-Prot protein database (Pundir *et al.* 2016) using BLASTP with an e-value cutoff of 10^−20^. In this way, putative functions were assigned to gene annotations.

To assess the quality and completeness of annotations in the *G. affinis* genome in an evolutionary context, we ran Benchmarking Universal Single-Copy Orthologs (BUSCO) v 2.0.1 (Simão *et al.* 2015; File S7). BUSCO uses a set of genes from major lineages (*e.g.*, 66 species of fish with a sequenced genome, actinopterygii_odb9; Malmstrøm *et al.* 2017) that are orthologous groups with genes present as single-copy orthologs in at least 90% of the species in the group. Therefore, we quantitatively checked for expected gene content while allowing for rare gene duplications or losses. The BUSCO pipeline incorporates AUGUSTUS v3.2.2 (Stanke and Morgenstern 2005), BLAST+ v2.6.0 (Camacho *et al.* 2009), and HMMER v3.1b2 (Finn *et al.* 2011).

### Noncoding RNA prediction

Transfer RNAs in the *G. affinis* genome were predicted using tRNAscan-SE 2.0 (Lowe and Chan 2016; File S8). The training set used for training the covariance model employed by tRNAscan-SE 2.0 was comprised of eukaryotic tRNAs. Ten of the predicted tRNAs decoding for amino acids were selected randomly and their sequences were searched against databases of tRNAs, GtRNAdb (Chan and Lowe 2016) and tRNAdb (Juhling *et al.* 2009). Lastly, we compared the predicted classes of tRNAs in the *G. affinis* genome with tRNAs reported in the genomes of *X. maculatus* (Schartl *et al.* 2013), *P. reticulata* (Künstner *et al.* 2016), and *O. latipes* (Chan and Lowe 2016).

Homology-based prediction was used to detect rRNAs (ribosomal RNA), snRNAs (small nuclear RNA), snoRNAs (small nucleolar RNA) and miRNAs (microRNAs) in the *G. affinis* genome (File S8). ncRNAs from *O. latipes*, *X. maculatus*, *G. aculeatus*, and *D. rerio* were downloaded from Ensemble (http://useast.ensembl.org/info/data/ftp/index.html) to create separate multispecies ncRNA databases for the rRNAs, snRNAs, snoRNAs and miRNAs. The following versions of fish databases were downloaded from Ensemble: BROAD S1 (*Gasterosteus aculeatus*), Xipmac4.4.2 (*Xiphophorus maculatus*), HdrR (*Oryzias latipes*) and GRCz10 (*Danio rerio*). These databases were used as queries by BLASTN to predict homologous rRNAs, snRNAs, snoRNAs and miRNAs in the *G. affinis* genome and the duplicates were removed from the output files. An e-value cutoff of less than 10^−5^ was employed to filter out significant hits. miRNAs were identified using the RNAfold program of the Vienna RNA package (v2.4.3) of MiRscan (http://genes.mit.edu/mirscan). miRNA sequences corresponding to structures having a minimum free energy of < -20kcal/mol were retained in the final output.

### Transposable elements

We compared the proportion and composition of transposable elements (TEs) in *X. maculatus*, *P. reticulata*, *O. latipes*, and *G. affinis* genomes. To determine the repeat diversity in the assembly, we used RepeatModeler (Smit and Hubley 2008–2015) with default parameters to identify and build a library containing transposable elements, simple repeats and low complexity regions (this library contained 737 consensus sequences). We also classified Miniature Inverted Repeat Transposable Elements (MITEs), which are not found with RepeatModeler, using MITE-Hunter (Han and Wessler 2010). As MITEs are non-autonomous sequences that lack protein-coding regions and distinctive features such as poly A tracts, MITE-Hunter may detect false positives. To avoid false positives, we used various MITE-specific criteria: 1) Terminal Inverted Repeats (TIRs) and Target Site Duplications (TSDs) identified by multiple sequence alignment (MSA) while flanking regions were divergent; 2) the high repetition of MITEs in their host genome; 3) and the identification of associated autonomous DNA transposons. Thus, we performed the following analyses for each consensus sequence: TSD identification in the MSA file, use of BlastN2 and RNAfold (Lorenz *et al.* 2011) to help identify the TIRs and TSDs, family identification using CENSOR (Kohany *et al.* 2006), and copy number estimation in the assembly via BLAST analysis.

We used RepeatMasker 4.0.0 (Smit, AFA, Hubley, R & Green, P. *RepeatMasker Open-4.0*) with the -lib option to specify the *G. affinis*-specific library to estimate the number of copies of each class and transposable element family as well as the coverage in the assembly.

### Data availability

Raw reads have been deposited in the NCBI Sequence Read Archive (SRR5601730 for the Nebraska fish/HiRise assembly, and SRR5601729 for the Japanese fish/Meraculous assembly). This Whole Genome Shotgun project has been deposited at DDBJ/ENA/GenBank under the accession NHOQ00000000. The version described in this paper is version NHOQ01000000. The genome sequence, annotations, and aligned reads (in BAM format) are available at gambusia.genetics.uga.edu. Figure S1 compares the size distribution of library inserts in the Meraculous and HiRise assemblies. Figure S2 shows the frequency of kmers at each kmer length. Figure S3 shows the distribution of scaffold lengths in the HiRise assembly. Figure S4 shows the cumulative percent of the assembly for a given scaffold size in the Meraculous and HiRise assemblies. Table S1 presents a detailed list of the number of copies and percent of the assembly of transposons and repeatable elements. Files S1-S4 contain the MAKER submission script, executable file (maker_exe.ctl), specifications for downstream filtering of BLAST and Exonerate alignments (maker_bopts.ctl), and primary configuration of MAKER specific options (maker_opts.ctl), respectively. File S5 contains the commands for training SNAP. File S6 contains the submission script for BLAST comparing Gambusia with related fish. File S7 contains the submission script for BUSCO. File S8 contains the submission script for predicting ncRNAs. File S9 contains the Illumina reads aligned to the reference in BAM format. The sequence and structure of tRNAs can be found in Files S10 and S11, respectively. File S12 contains the rRNA, snRNA, snoRNA, and miRNA sequences. Supplemental material available at Figshare: https://doi.org/10.25387/g3.6157706.

## Results

### Assembly

We sequenced the whole genome of the mosquitofish, *Gambusia affinis*, using one male fish from the invasive range in Japan for the initial shotgun sequencing and a second male fish from the invasive range in Nebraska, USA for the HiRise sequencing. We produced a 598.7 Mb genome assembly with 54.9× coverage on average (File S9). Using kmer analysis, we estimate the size of the genome to be 683 Mbp with Meraculous’ mercounter and 759 Mbp with Dovetail’s mercounter, with 18.5% of the genome repetitive. The N50 contig size was 17.6 kb and scaffold size was 6.65 Mb (Table 1; Figs. S1 and S3). This was a large improvement over the initial shotgun assembly, which had 24× coverage and contig and scaffold sizes of 13.9 kb and 31 kb, respectively (Table 1; Fig S4). In addition to the increase in scaffold size, we also had a large increase in contiguity, with the number of scaffolds above the median length decreasing from 5,240 in the Meraculous assembly to 26 in the HiRise assembly (Table 1). The overall number of scaffolds decreased from 38,526 to 2,943 (Table 1).

**Table 1:** Quality statistics of initial shotgun sequencing assembled by Meraculous and final assembly by HiRise

	Meraculous Assembly	Dovetail HiRise Assembly
Total length	594.6 Mb	598.7 Mb
Scaffold N50	31 kb	6.65 Mb
Scaffold N90	7 kb	914 kb
Scaffold L50	5,240 scaffolds	26 scaffolds
Scaffold L90	20,613 scaffolds	117 scaffolds
Longest scaffold	324,444	24,339,338
Number of scaffolds	38,526	2,943
Number of scaffolds >1 kb	38,519	2,940
Contig N50	13.9 kb	17.6 kb
Contig N90	3.56 kb	4.23 kb
Contig L50	12,100 contigs	9,490 contigs
Contig L90	44,284 contigs	35,674 contigs
Number of gaps >= 100 bp*^a^*	18,145	40,532
Percent of genome in gaps	0.972%	1.34%

a HiRise arbitrarily sizes gaps to 100 Ns.

### Gene prediction and annotation

The final annotation set of the *G. affinis* genome from the MAKER annotation pipeline contained 21,163 predicted genes (Table 2), fewer than closely related species (*P. reticulata*, *X. maculatus* and *O. latipes*). BLASTP analyses revealed 20,511 (97%), 19,904 (94%) and 18,880 (89%) of predicted *G. affinis* genes had significant hits to *P. reticulata*, *X. maculatus* and *O. latipes* respectively. Average gene, exon, and intron lengths are shorter in *G. affinis* when compared to closely related organisms, but average coding sequence length and the number of exons per gene are similar (Table 2). A total of 17,565 gene annotations were assigned putative functions through BLASTP analyses.

**Table 2:** Comparison of genes predicted in *Gambusia affinis* from BLAST to genome annotations for *Poecilia reticulate* (guppy), *Xiphophorus maculatus* (platyfish), and *Oryzias latipes* (medaka) from NCBI ‬

	*G. affinis*	*P. reticulata**^a^*	*X. maculatus**^b^*	*O. latipes**^c^*
Number of protein-encoding genes	21,144	22,982	22,082	22,658
Mean gene length (bp)	13,510	18,441	15,702	16,221
Mean CDS length (bp)	1,827	2,175	1,714	1,893
# of *G. affinis* BLASTP Hits	—	20,511	19,904	18,880
Number of exons	236,097	276,363	227,016	258,916
Mean exon length (bp)	164	267	189	260
Mean number of exons per gene	11.2	12.9	10.6	11.0
Number of introns	214,953	248,065	205,251	230,293
Mean intron length (bp)	1,151	2,000	1,500	1,726

a http://www.ncbi.nlm.nih.gov/genome/annotation_euk/Poecilia_reticulata/100/

b http://www.ncbi.nlm.nih.gov/genome/annotation_euk/Xiphophorus_maculatus/101/

c http://www.ncbi.nlm.nih.gov/genome/annotation_euk/Oryzias_latipes/101/

In 66 sequenced fish genomes, 4584 genes are found as single copy in at least 90% of these species. In the *G. affinis* genome, 95.5% (4379) of these 4584 genes had “complete” orthologs, defined as genes that scored within the expected range and were within the expected length. Of these, 93.4% of the total were found in single copy and 2.1% were duplicated. About 2.6% (120) of genes were “fragmented”, meaning that there was a significant match to a gene within the *G. affinis* genome, but the length was outside of two standard deviations of the BUSCO group mean length, either because the gene is only partially present or indicating a problem with the genome assembly. The last 1.9% (85 genes) had no significant matches, indicating that the ortholog is missing or highly divergent, the gene prediction failed, or those genes are incorrectly assembled.

### ncRNA prediction

1769 tRNAs were detected by tRNAscan-SE 2.0 in total (see Files S10 and S11 for the sequence and structure of tRNAs), out of which 260 were found to decode for amino acids, including a single tRNA which decodes for selenocysteine. 22 tRNAs had undetermined isotypes. Related species had more tRNAs decoding amino acids and tRNAs with undetermined isotypes (Table 3). 1453 tRNAs were detected as pseudogenes with poor primary/secondary structures, more than in *O. latipes* but fewer than in *P. reticulata*. These were found to have a low Infernal as well as Isotype score in the predicted output from tRNAscan-SE 2.0. Thirty-four tRNAs were chimeric with mismatched isotypes, meaning they have specific identity elements in their bodies which are recognized by specific tRNA synthetases, but they code for mRNAs corresponding to different amino acids due to point mutations in their anticodon sequence. Hence, there exists a disagreement in their functional classification, with predicted isotype based on the anticodon sequence and another predicted by the isotype-specific covariance model (Lowe and Chan 2016).

**Table 3:** The number of tRNAs predicted in the *Gambusia affinis* genome compared to *Xiphophorus maculatus* (platyfish), *Poecilia reticulata* (guppy), and *Oryzias latipes* (medaka)

	*G. affinis*	*P. reticulata*	*X. maculatus*	*O. latipes*
tRNAs decoding standard 20 AA	260	439	535	726
Selenocysteine tRNAs	1	3	–	4
Possible suppressor tRNAs	0	1	–	2
tRNAs with undetermined or unknown isotypes	22	65	–	603
Predicted pseudogenes	1453	4186	–	497

284 tRNAs had introns, out of which 257 were predicted to be pseudogenes, two were chimeras, and 25 decoded for the twenty standard amino acids. No suppressor tRNAs were found in the analysis. The subset of predicted tRNAs decoding for amino acids were also predicted in a large number of other species in both GtRNAdb and tRNAdb. 665 miRNAs, 4 rRNAs, 50 snRNAs, and 164 snoRNAs were predicted in the *G. affinis* genome (Table 4; File S12). Compared to other fish with sequenced genomes, including *X. maculatus*, *O. latipes*, *G. aculeatus*, and *D. rerio*, *G. affinis* had the highest number of miRNAs predicted, but fewest rRNAs, snRNAs, and snoRNAs.

**Table 4:** The number of noncoding RNAs predicted in *Gambusia affinis* compared to *Xiphophorus maculatus* (platyfish), *Oryzias latipes* (medaka), *Gasterosteus aculeatus* (stickleback), and *Danio rerio* (zebrafish)

	*G. affinis*	*X. maculatus*	*O. latipes*	*G. aculeatus*	*D. rerio*
miRNA	665	342	366	504	440
rRNA	4	6	57	416	1579
snRNA	50	–	76	366	1287
snoRNA	164	–	225	297	305

### Transposable elements

MITE-hunter found 170 consensus elements and, of these, 102 consensus elements were from 24 families and the other 68 were from singlet families. After further sequence analyses, 35 sequences were added in the repeat library. Twenty of these 205 total repetitive sequences were found to be very conserved relative to the *X. maculatus* genome (full length sequence, >90% of identity).

Non-genic repeats accounted for ∼20% of the assembly with the great majority (∼17.7%) coming from TEs (Table 5, Table S1). Among TEs, DNA transposons are the most abundant class, with the TcMariner and hAT families particularly abundant. The *G. affinis* assembly is less repetitive than other sequenced poeciliid genomes (from the *Xiphophorus* genus, Shen *et al.* 2016), primarily due to higher contents of TcMariner and hAT families in other fish genomes.

**Table 5:** Number and percent of transposons and other repeats in the *Gambusia affinis* genome

Classification	Number of copies	Percentage of assembly
DNA Transposons	318,331	9.361
LTR Retrotransposons	12,602	0.379
LINE Retrotransposons	50,048	1.401
SINE Retrotransposons	16,609	0.427
Unknown	198,564	6.23
***Total transposable elements****^a^*	***596,154***	***17.799***
Low complexity regions*^b^*	33,073	0.255
Satellites*^c^*	4,914	0.229
Microsatellites	219,965	1.431
**Total**	**854,106**	**19.714**

a Includes DNA transposons, LTR, LINE, SINE retrotransposons and unknown.

b Regions composed of a single or two nucleotides, *e.g.*: A-rich, GA-rich, C-rich.

c Duplications of complex sequences 100-200 bp long.

## Discussion

We sequenced and assembled the genome of the mosquitofish, *Gambusia affinis*, using short read Illumina data from paired-end and *in vitro* proximity-ligation Chicago libraries. The resulting genome assembly had high coverage, improved contigs, and long scaffold sizes compared to other assemblies that used Illumina mate-paired libraries (*Poecilia reticulata*; Künstner *et al.* 2016), assemblies that utilized Roche 454 long insert sequencing (*Xiphophorus maculatus*; Schartl *et al.* 2013), or multiple types of sequencing reads, including PacBio reads (Pootakham *et al.* 2017). The Chicago library improves the scaffold contiguity because it provides links between genomic regions hundreds of kb apart and uses information about proximity ligation libraries to obtain a highly continuous genome assembly (Putnam *et al.* 2016). The result is a high-quality genome sequence composed of 26 (N50) scaffolds, just more than the haploid number of chromosomes (n = 24; Chen and Ebeling 1968). Our genome assembly was 598.7 Mbp, slightly shorter than the Meraculous kmer estimate of 683 Mbp and the Dovetail kmer estimate of 759 Mbp. The kmer estimates differ because the Dovetail kmercounter used untrimmed reads, and therefore had deeper coverage and better discrimination of homozygotes and heterozygotes, and fully counted the repeats without a maximum copy count. Previous estimates of n = 0.74 to 0.76 pg (724 to 743 Mbp) from white blood cells from 50 native fish, averaged between males and females (Tiersch *et al.* 1989) and n = 0.695 to 0.855 pg (680 to 836 Mbp) from blood of two invasive fish, where the sex was not recorded (Jianxun *et al.* 1991), have been reported using flow cytomery. *G. affinis* has dimorphic (WZ) sex chromosomes, where males are homomorphic ZZ and females are heteromorphic ZW, and the W chromosome is the single largest metacentric chromosome and Zs are the smallest acrocentric chromosomes (Black and Howell 1979; Chen and Ebeling 1968). Therefore, we expect a smaller assembly size for our male fish than flow cytometry estimates that average both females and males (Jianxun *et al.* 1991; Tiersch *et al.* 1989).

We found that approximately 90% or more of the genes in *G. affinis* had hits in the Poeciliid family and in other fish species. Similarly, the majority of genes in the BUSCO gene set were detected in single copy in the *G. affinis* genome, indicating that the *G. affinis* genome was largely complete. The number of TEs reported here comprise slightly less of the *G. affinis* genome than other Poeciliidae genomes, which average ∼21% TEs (Shen *et al.* 2016), but this difference is well within the range of expected variation among species and accuracy of the estimated scaffolds, especially when considering the variance in approaches used for scaffolding.

Although we find fewer tRNAs, rRNAs, snRNAs and snoRNAs than in related species, we have high confidence in the predicted ncRNAs we report because we used conservative cutoffs to reduce false positives. We find similar relative abundances of each type of ncRNA as in related fish species.

*G. affinis* is a model organism in diverse fields of ecology and evolution, such as life-history evolution (Haynes and Cashner 1995), behavior (Cote *et al.* 2010), and biological invasions (Rehage *et al.* 2005). The genome assembly and annotations we have created will be a useful resource for those interested in mapping a genetic architecture to traits of interest in this species. In addition, this genome serves as a resource in comparative genomics among Poecilids and teleosts.

## References

[bib1] Allen G. C. Flores-Vergara M. A. Krasynanski S. Kumar S. Thompson W. F., 2006 A modified protocol for rapid DNA isolation from plant tissues using cetyltrimethylammonium bromide. Nat. Protoc. 1: 2320–2325. 10.1038/nprot.2006.384 17406474

[bib2] Basolo A. L., 1990 Female preference for male sword length in the green swordtail, *Xiphophorus helleri* (Pisces: Poeciliidae). Anim. Behav. 40: 332–338. 10.1016/S0003-3472(05)80928-5

[bib3] Black D. A. Howell W. M., 1979 The North American mosquitofish, *Gambusia affinis*: A unique case in sex chromosome evolution. Copeia 1979: 509–513. 10.2307/1443231

[bib4] Bolger A. M. Lohse M. Usadel B., 2014 Trimmomatic: a flexible trimmer for Illumina sequence data. Bioinformatics 30: 2114–2120. 10.1093/bioinformatics/btu170 24695404 PMC4103590

[bib5] Camacho C. Coulouris G. Avagyan V. Ma N. Papadopoulos J., 2009 BLAST+: architecture and applications. BMC Bioinformatics 10: 421 10.1186/1471-2105-10-421 20003500 PMC2803857

[bib6] Campbell M. S. Holt C. Moore B. Yandell M., 2014 Genome annotation and curation using MAKER and MAKER‐P. Current Protocols in Bioinformatics. 48:4.11.1–39. 10.1002/0471250953.bi0411s48 PMC4286374 25501943

[bib7] Chan P. P. Lowe T. M., 2016 GtRNAdb 2.0: An expanded database of transfer RNA genes identified in complete and draft genomes. Nucleic Acids Res. 44: D184–D189. 10.1093/nar/gkv1309 26673694 PMC4702915

[bib8] Chapman J. A. Ho I. Sunkara S. Luo S. Schroth G. P., 2011 Meraculous: de novo genome assembly with short paired-end reads. PLoS One 6: e23501 10.1371/journal.pone.0023501 21876754 PMC3158087

[bib9] Chen T. R. Ebeling A. W., 1968 Karyological evidence of female heterogamety in the mosquitofish, *Gambusia affinis*. Copeia 1968: 70–75. 10.2307/1441552

[bib10] Constanz G. D., 1975 Behavioral ecology of mating in the male gila topminnow, *Poecilipsis occidentalis* (Cyprinodontiformes: Poeciliidae). Ecology 56: 966–973. 10.2307/1936307

[bib11] Cote J. Fogarty S. Weinersmith K. Brodin T. Sih A., 2010 Personality traits and dispersal tendency in the invasive mosquitofish (*Gambusia affinis*). Proc. R. Soc. Lond. B Biol. Sci. 277: 1571–1579. 10.1098/rspb.2009.2128 PMC2871838 20071380

[bib12] Farr J. A., 1980 Social behavior patterns as determinants of reproductive success in the guppy, *Poecilia reticulata* Peters (Pisces: Poeciliidae) an experimental study of the effects of intermale competition, female choice, and sexual selection. Behaviour 74: 38–90. 10.1163/156853980X00311

[bib13] Finn R. D. Clements J. Eddy S. R., 2011 HMMER web server: interactive sequence similarity searching. Nucleic Acids Res. 39: W29–W37. 10.1093/nar/gkr367 21593126 PMC3125773

[bib14] Fraser B. A. Weadick C. J. Janowitz I. Rodd F. H. Hughes K. A., 2011 Sequencing and characterization of the guppy (*Poecilia reticulata*) transcriptome. BMC Genomics 12: 202 10.1186/1471-2164-12-202 21507250 PMC3113783

[bib50] Han Y. Wessler S. R., 2010 MITE-Hunter: A program for discovering miniature inverted-repeat transposable elements from genomic sequences. Nucleic Acids Res. 38: e199 10.1093/nar/gkq862 20880995 PMC3001096

[bib15] Haynes J. L. Cashner R. C., 1995 Life-history and popluation-dynamics of the western mosquitofish - A comparison of natural and introduced populations. J. Fish Biol. 46: 1026–1041. 10.1111/j.1095-8649.1995.tb01407.x

[bib16] Howard H., 1920 Malaria Control in Rural Communities by Anti-Mosquito Measures. South. Med. J. 13: 260–266. 10.1097/00007611-192004000-00007

[bib17] Howard L. O., 1910 *Preventive and Remedial Work Against Mosquitoes*. US Department of Agriculture, Department of Agriculture, Washington, D.C 10.5962/bhl.title.65046

[bib18] Jianxun C. Xiuhai R. Qixing Y., 1991 Nuclear DNA content variation in fishes. Cytologia (Tokyo) 56: 425–429. 10.1508/cytologia.56.425

[bib19] Juhling F. Morl M. Hartmann R. K. Sprinzl M. Stadler P. F., 2009 tRNAdb 2009: Compilation of tRNA sequences and tRNA genes. Nucleic Acids Res. 37: D159–D162. 10.1093/nar/gkn772 18957446 PMC2686557

[bib20] Kasahara M. Naruse K. Sasaki S. Nakatani Y. Qu W., 2007 The medaka draft genome and insights into vertebrate genome evolution. Nature 447: 714–719. 10.1038/nature05846 17554307

[bib48] Kohany O. Gentles A. J. Hankus L. Jurka J., 2006 Annotation, submission and screening of repetitive elements in Repbase: RepbaseSubmitter and Censor. BMC Bioinformatics 7: 1–7. 10.1186/1471-2105-7-474 17064419 PMC1634758

[bib21] Korf I., 2004 Gene finding in novel genomes. BMC Bioinformatics 5: 59 10.1186/1471-2105-5-59 15144565 PMC421630

[bib22] Krumholz L. A., 1948 Reproduction in the western mosquitofish, *Gambusia affinis affinis* (Baird & Girard), and its use in mosquito control. Ecol. Monogr. 18: 1–43. 10.2307/1948627

[bib23] Künstner A. Hoffmann M. Fraser B. A. Kottler V. A. Sharma E., 2016 The Genome of the Trinidadian Guppy, *Poecilia reticulata*, and Variation in the Guanapo Population. PLoS One 11: e0169087 10.1371/journal.pone.0169087 28033408 PMC5199103

[bib24] Lamatsch D. Steinlein C. Schmid M. Schartl M., 2000 Noninvasive determination of genome size and ploidy level in fishes by flow cytometry: Detection of triploid *Poecilia formosa*. Cytometry 39: 91–95. 10.1002/(SICI)1097-0320(20000201)39:2<91::AID-CYTO1>3.0.CO;2-4 10679726

[bib25] Langerhans R. B. Layman C. A. DeWitt T. J., 2005 Male genital size reflects a tradeoff between attracting mates and avoiding predators in two live-bearing fish species. Proc. Natl. Acad. Sci. USA 102: 7618–7623. 10.1073/pnas.0500935102 15894618 PMC1140428

[bib26] Lever C., 1996 Naturalized Fishes of the World, Academic Press, New York.

[bib27] Lloyd L. Arthington A. Milton D., 1986 The mosquitofish—a valuable mosquito-control agent or a pest, The ecology of exotic animals and plants some Australian case histories, edited by Kitching R. L., Wiley, Brisbane.

[bib49] Lorenz R. Bernhart S. H. Honer zu Siederdissen C. Tafer H. Flamm C. Stadler P. F. Hofacker I. L., 2011 ViennaRNA Package 2.0. Algorithms for Molecular Biology 6: 1–14. 10.1186/1748-7188-6-26 PMC3319429 22115189

[bib28] Lowe T. M. Chan P. P., 2016 tRNAscan-SE on-line: integrating search and context for analysis of transfer RNA genes. Nucleic Acids Res. 44: W54–W57. 10.1093/nar/gkw413 27174935 PMC4987944

[bib29] Malmstrøm M. Matschiner M. Tørresen O. K. Jakobsen K. S. Jentoft S., 2017 Whole genome sequencing data and de novo draft assemblies for 66 teleost species. Sci. Data 4: 160132 10.1038/sdata.2016.132 28094797 PMC5240625

[bib30] Pollux B. Pires M. Banet A. Reznick D., 2009 Evolution of placentas in the fish family Poeciliidae: an empirical study of macroevolution. Annu. Rev. Ecol. Evol. Syst. 40: 271–289. 10.1146/annurev.ecolsys.110308.120209

[bib31] Pootakham W. Sonthirod C. Naktang C. Ruang-Areerate P. Yoocha T., 2017 *de novo* hybrid assembly of the rubber tree genome reveals evidence of paleotetraploidy in *Hevea* species. Sci. Rep. 7: 41457 10.1038/srep41457 28150702 PMC5288721

[bib32] Pundir S. Martin M. J. O’Donovan C., 2016 UniProt tools. Curr. Protoc. Bioinformatics 45: D158–D169. 10.1002/0471250953.bi0129s53 PMC4941944 27010333

[bib33] Putnam N. H. O’Connell B. L. Stites J. C. Rice B. J. Blanchette M., 2016 Chromosome-scale shotgun assembly using an in vitro method for long-range linkage. Genome Res. 26: 342–350. 10.1101/gr.193474.115 26848124 PMC4772016

[bib34] Pyke G. H., 2008 Plague minnow or mosquito fish? A review of the biology and impacts of introduced *Gambusia* species. Annu. Rev. Ecol. Evol. Syst. 39: 171–191. 10.1146/annurev.ecolsys.39.110707.173451

[bib35] Rehage J. Barnett B. Sih A., 2005 Foraging behaviour and invasiveness: Do invasive *Gambusia* exhibit higher feeding rates and broader diets than their noninvasive relatives? Ecol. Freshwat. Fish 14: 352–360. 10.1111/j.1600-0633.2005.00109.x

[bib36] Reznick D. Endler J. A., 1982 The impact of predation on life history evolution in Trinidadian guppies (*Poecilia reticulata*). Evolution 36: 160–177. 10.1111/j.1558-5646.1982.tb05021.x 28581096

[bib37] Sakai A. K. Allendorf F. W. Holt J. S. Lodge D. M. Molofsky J., 2001 The population biology of invasive species. Annu. Rev. Ecol. Syst. 32: 305–332. 10.1146/annurev.ecolsys.32.081501.114037

[bib38] Schartl M. Walter R. B. Shen Y. Garcia T. Catchen J., 2013 The genome of the platyfish, Xiphophorus maculatus, provides insights into evolutionary adaptation and several complex traits. Nat. Genet. 45: 567–572. 10.1038/ng.2604 23542700 PMC3677569

[bib39] Shen Y. Chalopin D. Garcia T. Boswell M. Boswell W., 2016 *X. couchianus* and *X. hellerii* genome models provide genomic variation insight among *Xiphophorus* species. BMC Genomics 17: 37 10.1186/s12864-015-2361-z 26742787 PMC4705583

[bib40] Simão F. A. Waterhouse R. M. Ioannidis P. Kriventseva E. V. Zdobnov E. M., 2015 BUSCO: assessing genome assembly and annotation completeness with single-copy orthologs. Bioinformatics 31: 3210–3212. 10.1093/bioinformatics/btv351 26059717

[bib41] Smit A. Hubley R., 2008–2015 RepeatModeler open-1.0. Available at: http://www.repeatmasker.org. Accessed: March 17, 2016.

[bib42] Stanke M. Morgenstern B., 2005 AUGUSTUS: a web server for gene prediction in eukaryotes that allows user-defined constraints. Nucleic Acids Res. 33: W465–W467. 10.1093/nar/gki458 15980513 PMC1160219

[bib43] Tiersch T. R. Chandler R. W. Wachtel S. S. Elias S., 1989 Reference standards for flow cytometry and application in comparative studies of nuclear DNA content. Cytometry 10: 706–710. 10.1002/cyto.990100606 2582960

[bib44] Tobler M. Schlupp I. Garcia de León F. J. Glaubrecht M. Plath M., 2007 Extreme habitats as refuge from parasite infections? Evidence from an extremophile fish. Acta Oecol. 31: 270–275. 10.1016/j.actao.2006.12.002

[bib45] Tobler M. Schlupp I. Heubel K. U. Riesch R. Garcia de León F. J., 2006 Life on the edge: Hydrogen sulfide and the fish communities of a Mexican cave and surrounding waters. Extremophiles 10: 577–585. 10.1007/s00792-006-0531-2 16788733

[bib46] Trexler J. C. Travis J., 1990 Phenotypic plasticity in the sailfin molly, *Poecilia latipinna* (Pisces: Poeciliidae). I. Field experiments. Evolution 44: 143–156. 10.1111/j.1558-5646.1990.tb04285.x 28568197

[bib47] Volff J.-N. Schartl M., 2001 Variability of genetic sex determination in poeciliid fishes. Genetica 111: 101–110. 10.1023/A:1013795415808 11841158

